# A meta-analysis of randomized controlled trials comparing breast-conserving surgery and mastectomy in terms of patient survival rate and quality of life in breast cancer

**DOI:** 10.1093/intqhc/mzae043

**Published:** 2024-05-16

**Authors:** Shuangjian Li, Xin Li, Dan Li, Qian Zhao, Liping Zhu, Tao Wu

**Affiliations:** Department of Breast Surgery, The Affiliated Cancer Hospital of Xinjiang Medical University, Xinjiang Key Laboratory of Oncology, Urumqi, Xinjiang 830011, China; Department of Operating Room, The First People’s Hospital of Urumqi Economic and Technological Development Zone, Toutunhe District, Urumqi 830011, Xinjiang, China; Department of Breast Surgery, The Affiliated Cancer Hospital of Xinjiang Medical University, Xinjiang Key Laboratory of Oncology, Urumqi, Xinjiang 830011, China; Department of Breast Surgery, The Affiliated Cancer Hospital of Xinjiang Medical University, Xinjiang Key Laboratory of Oncology, Urumqi, Xinjiang 830011, China; Department of Breast Surgery, The Affiliated Cancer Hospital of Xinjiang Medical University, Xinjiang Key Laboratory of Oncology, Urumqi, Xinjiang 830011, China; Department of Breast Surgery, The Affiliated Cancer Hospital of Xinjiang Medical University, Xinjiang Key Laboratory of Oncology, Urumqi, Xinjiang 830011, China

**Keywords:** breast neoplasms, mastectomy, breast-conserving, quality of life, survival rate

## Abstract

The study aimed to assess the effects of breast-conserving surgery (BCS) versus mastectomy on survival and quality of life in Stages I, II, and III breast cancer, providing solid evidence for clinical decisions. We conducted a meta-analysis of randomized controlled trials on breast cancer treatments, searching databases such as PubMed and the Cochrane Library to compare BCS, and mastectomy’s effects on survival and quality of life. A combined total of 16 734 patients in the control group and 17 435 patients in the experimental group were included in this analysis. This meta-analysis used RevMan 5.3 (Cochrane Collaboration, Copenhagen, Denmark) software for analysis. Our meta-analysis of 34 169 patients from 11 studies showed that BCS significantly reduced the overall recurrence rate at a median follow-up of 29 months, with a mean difference of 1.27 and a 95% confidence interval of 1.19–1.36, strongly supporting its effectiveness (*P* *<* .00001). Furthermore, our analysis found no significant increase in 5-year local recurrence rates for BCS versus mastectomy, indicating its long-term effectiveness with a mean difference of 1.13 (95% confidence interval: [1.03, 1.24], *P = *.01). Additionally, there was a notable decrease in tissue ischaemic necrosis among patients who had received BCS, with a mean difference of 0.37 (95% confidence interval: [0.33, 0.42], *P < *.00001), underscoring its benefits and long-term viability. BCS resulted in fewer cases of tissue ischaemic necrosis and higher body image scores compared with mastectomy, suggesting that it is a preferable option for better cosmetic outcomes and potentially favourable effects on prognosis and quality of life.

## Introduction

Breast cancer is the most common malignancy among women worldwide, with a total of 1.7 million new cases reported by 2012, accounting for 25% of all malignancies in women [1]. In 2011, China had 248 000 new cases, contributing to 12% of global breast cancer incidents. The incidence rate in urban areas was higher than that in rural areas [2]. With advancements in diagnostic and therapeutic methods, many breast cancer cases are now detected at an early stage, leading to increased survival rates and a growing number of breast cancer survivors [3]. Consequently, patients’ quality of life has gained prominence as a vital indicator for assessing treatment and rehabilitation outcomes [2].

The breast is a crucial secondary sexual characteristic in women, and surgical interventions undoubtedly have irreversible negative impacts on patients’ physical, psychological, and social aspects, thus affecting their overall quality of life [4]. Research into the impact of different surgical approaches on quality of life has become increasingly important. Clinical studies with 20 years of follow-up have confirmed that breast-conserving surgery (BCS) combined with radiation therapy achieves comparable survival rates to mastectomy in early-stage breast cancer cases. Hence, BCS has become a standard procedure [5–7]. This method is less invasive, offers better cosmetic outcomes, and preserves better physical function. However, international research suggests that although patients who have undergone BCS show significant advantages in body image aspects (including attractiveness, appearance, confidence, scars, and cosmetic outcomes), there may not be a substantial improvement in the overall quality of life [1]. Some studies [3, 8] even report that patients undergoing BCS with radiation therapy may experience more concerns about tumour recurrence, breast pain, and limited upper limb functionality, leading to a lower quality of life.

Recent studies examining the impact of different breast cancer surgical approaches on patients’ quality of life are scarce and yield varied conclusions [1, 9–12]. Although some clinical comparative studies have discussed the effects of BCS versus mastectomy on survival rates and quality of life among patients with breast cancer, there remains a need for updated systematic reviews encompassing recent, high-quality clinical research. Therefore, this study employs a meta-analysis to comprehensively evaluate the relevant research on the impact of BCS versus mastectomy on patient survival rates and quality of life. We hypothesize that BCS will show improved quality-of-life metrics without significantly compromising survival rates. This approach aims to explore in depth the features of breast cancer and primarily investigate the influence of these surgical approaches on patients, with the ultimate goal of assessing their clinical value for breast cancer prevention and treatment. Valuation of the clinical relevance of our findings by emphasizing that BCS, although not entirely eradicating the tumour, significantly reduces overall recurrence rates and the incidence of postoperative complications, such as tissue ischaemic necrosis. This insight is crucial for practitioners aiming to balance effective cancer treatment with the preservation of quality of life. Furthermore, we delve into the psychological and physical benefits of BCS, substantiated by higher postoperative body image scores, highlighting its value in holistic patient care. While the study focuses on Stages I–III, the impact on patients with Stage IV breast cancer remains an area of future research.

We hypothesize that BCS will show improved quality-of-life metrics without significantly compromising survival rates. This approach aims to deeply explore the features of breast cancer and primarily investigate the influence of these surgical approaches on patients, with the ultimate goal of assessing their clinical value for breast cancer prevention and treatment.

## Research methodology

### Literature search strategy

**Figure 1:**
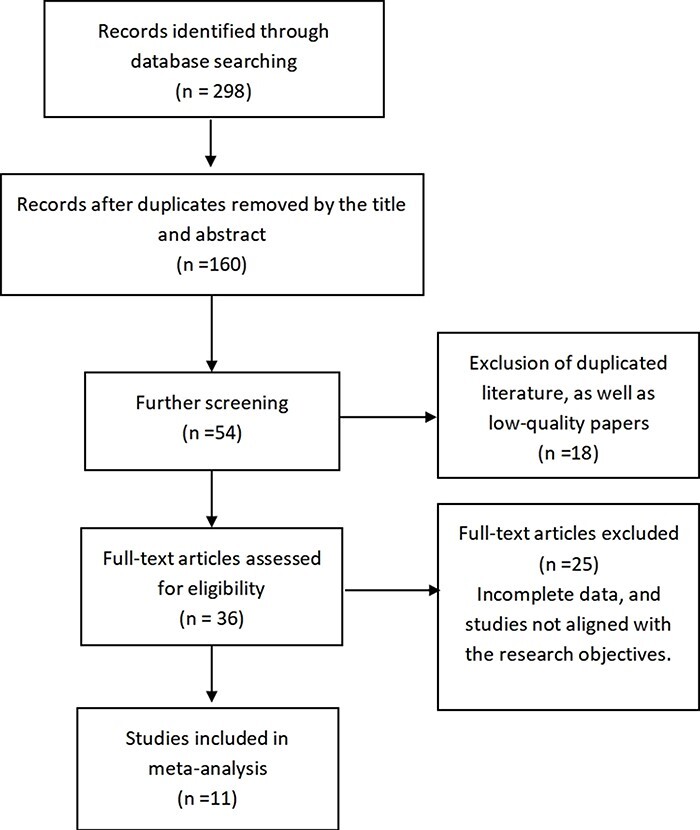
Literature selection flowchart.

Time frame: between database inception and 2023; databases searched (both Chinese- and English-language databases): PubMed, Embase, Web of Science, Cochrane Library. The specific search strategy was as follows: ‘Breast Neoplasms’[MeSH] AND ((‘Breast-Conserving Treatment’[MeSH] OR ‘Mastectomy’[MeSH]) AND (‘Survival Rate’[MeSH] OR ‘Quality of Life’[MeSH] AND ‘Outcome Assessment (Health Care)’[MeSH])) AND (‘Randomized Controlled Trials as Topic’[MeSH] OR ‘Clinical Trials as Topic’[MeSH]) AND (‘Meta-Analysis as Topic’[MeSH] OR ‘Systematic Reviews as Topic’[MeSH]) AND (‘Choice Behavior’[MeSH] OR ‘Treatment Outcome’[MeSH] OR ‘Long-Term Follow-Up Studies’[MeSH]) AND (‘Neoplasm Excision’[MeSH] OR ‘Neoplasm Recurrence, Local’[MeSH] AND ‘Risk Factors’[MeSH]) AND (‘Patient Satisfaction’[MeSH] AND ‘Case-Control Studies’[MeSH] OR ‘Cohort Studies’[MeSH]) AND (‘Antineoplastic Agents’[MeSH] AND ‘Comparative Study’[MeSH]) AND (‘Treatment Efficacy’[MeSH] AND ‘Review Literature as Topic’[MeSH]). Studies were included in English or with English translations, if available. Grey literature, such as unpublished theses, was excluded due to potential biases and variable quality. A preliminary search yielded 298 relevant articles. This number was reduced to 160 after a meticulous removal of duplicates based on title comparison. Subsequent in-depth examination of titles and abstracts led to an initial screening of 54 articles, focusing on their relevance and alignment with the research objectives. After further examination of the full texts, 18 articles were excluded due to their low methodological quality or because their focus did not align precisely with the research objectives, such as by diverging from the central topic of comparing BCS with mastectomy. This refined the selection to 36 articles. An additional rigorous round of full-text review was conducted, during which 25 articles were excluded because they contained incomplete data, had small sample sizes, or lacked necessary statistical information. Ultimately, 11 English-language articles that met all the stringent quality and relevance criteria were included in the analysis. Each step of this meticulous exclusion process is detailed in Fig. 1 to provide transparency and reproducibility. All surgeries were conducted in a sterile operating room environment with the patient under general anaesthesia. High-definition cameras and monitors were utilized for visualization in cases where endoscopic or minimally invasive approaches were employed. Standard surgical instruments were used, and all procedures were conducted by board-certified surgeons who specialize in breast surgery.

### Inclusion and exclusion criteria for the literature

#### Inclusion and exclusion criteria

The inclusion criteria of the study included (i) women with Stages I–III breast cancer, (ii) pathologically confirmed invasive ductal carcinoma, and (iii) completed chemotherapy and/or radiation therapy.

#### Exclusion criteria

The exclusion criteria of the study included (i) patients with severe chronic or disabling diseases, such as diabetes, Parkinson’s disease, or hemiplegia, (ii) inability to comprehend or read due to intellectual or mental factors, (iii) patients with recurrence or metastasis during the follow-up period, (iv) patients with bilateral mastectomy and patients who underwent neoadjuvant therapy before surgery or did not receive adjuvant therapy after surgery, (v) patients with a history of breast cancer surgery, (vi) patients with other malignant tumours, (vii) patients with impaired heart, liver or kidney function, (viii) patients with coagulation disorders, (ix) patients with synchronous bilateral breast cancer, and (x) patients aged ≥70 years were specifically excluded from this meta-analysis due to the increased complexity of comorbid conditions prevalent in this age group, which could confound the outcomes and impair the clarity of the analysis regarding the direct impact of BCS versus mastectomy on survival rates and quality of life.

#### Outcome measures

The outcome measures were as follows: (i) surgical details in both groups—a comparison of the procedural aspects between BCS and mastectomy; (ii) an enumeration of possible complications, such as tissue ischaemic necrosis, seroma, hematoma, and surgical site infections; (iii) prognosis and survival: 5-year local recurrence and survival outcomes in both groups, analysing and evaluating prognosis; and (iv) quality-of-life assessment utilizing the European Organization for Research and Treatment of Cancer Quality of Life Questionnaire (QLQ), specifically the QLQ-BR23 for breast cancer and QLQ-C30 for patients with general cancer.

### Literature quality assessment

Two researchers independently conducted the assessment of literature quality, and the results were compared and discussed. The study employs the QLQ-BR23 and QLQ-C30 questionnaires, renowned for their comprehensive approach to evaluating the various dimensions of a patient’s life post-surgery. These scales measure a wide range of factors including sexual function, upper limb symptoms, and breast symptoms. In the event of a disagreement during the literature quality assessment process, the issue was resolved by consultation with a senior researcher who possesses >10 years of experience in oncology research. This senior researcher’s adjudication was considered final to ensure the integrity and reliability of the assessment. These questionnaires collectively assess various dimensions including body image, sexual functioning, symptoms, and future perspectives. The inclusion of these measures allows for a nuanced understanding of how each surgical option affects the patient’s daily living and psychological well-being. This comprehensive approach ensures that our analysis remains patient-centred, reflecting the real-world implications of the chosen surgical method. The Cochrane Risk of Bias assessment tool was used to evaluate the quality of randomized controlled trials, including seven domains. Each domain was evaluated as ‘low risk of bias’, ‘high risk of bias’, or ‘unclear’. When all criteria were fully met, the likelihood of bias was minimized and categorized as Level A; when some criteria were partially met, the likelihood of bias was moderate and categorized as Level B; when none of the criteria were met, the likelihood of bias was higher and categorized as Level C.

### Statistical methods

Meta-analysis was conducted using the analysis module of RevMan 5.3 (Cochrane Collaboration, Copenhagen, Denmark). Analysis statistics for relative risk and standardized mean difference were presented within a 95% confidence interval (CI). Before combining study results, the *I*-squared statistic and the chi-squared test for heterogeneity were used to assess statistical heterogeneity among included studies. Values of *I*^2^ > 50% or *P* < 0.10 were considered significant indicators of heterogeneity among studies. For overall relative risk or standardized mean difference scores, when significant heterogeneity was present, a random-effects model was used to calculate the 95% CI; otherwise, a fixed-effects model was applied. Sensitivity or funnel analyses were planned to assess the influence of individual studies on the overall meta-analysis outcome. The outcome evaluation indicators in this study were continuous variables, represented by variance or weighted variance and were also presented with a 95% CI. The methodology for this assessment involves a detailed meta-analysis of the scores obtained from the selected studies, providing a comprehensive overview of the impact of each surgical option on the patient’s quality of life. The methodological approach for this analysis involved aggregating and comparing the scores from various studies to derive meaningful insights into the long-term effects of surgical procedures on the patient’s well-being.

## Results

### Quality assessment of included studies

**Figure 2:**
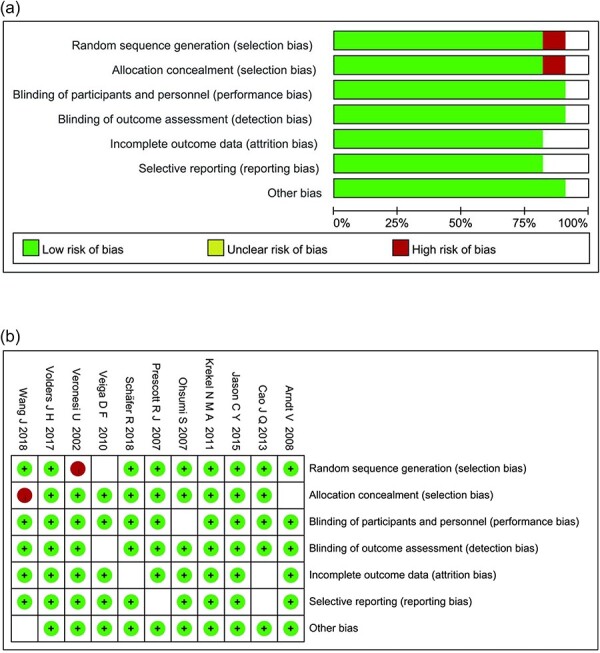
Literature quality assessment.

Among the 11 articles included in this study, 3 articles [13–15] were assessed as having a high level of methodological quality (Grade A), 6 articles [16–21] were of moderate quality (Grade B), and 2 articles [22, 23] were of lower quality (Grade C). Studies were graded ‘C’ primarily due to incomplete reporting of the patient follow-up. Nine articles provided detailed descriptions of their methods, and two articles reported concealed allocation methods. Additionally, 8 articles had comparable outcome measures, and all 11 articles were based on randomized controlled trial research designs (Fig. 2).

### Basic characteristics of included studies

A total of 11 randomized controlled trials and 1 self-matched before-and-after study were included, involving a combined total of 34 169 participants. Among them, 546 participants were involved in the self-matched before-and-after study. In addition, the ‘restrict to English language’ constraint may have excluded relevant studies conducted in non-English-speaking regions. Statistical analysis of different studies encompassed follow-up times and patient assessments of survival quality indicators. Notably, the assessment of patients’ quality of life (measured by the QLQ-BR23 and QLQ-C30 scales) was a primary focus for comparing life quality across studies, with only one study (Study 12) not including this comparison in its statistics. Among the 11 included studies, 1 received a C grade, 2 received an A grade, and the rest received a B grade. Furthermore, the analysis encompassed different adverse reactions and postoperative complications (Table 1).

**Table 1.** Characteristics of included studies.

References	Year	Location	Sample size	Intervention methods	Outcome	Follow-up time, years	Postoperative complications and adverse reactions	Comparison of quality-of-life assessments (QLQ-BR23 scale scores and QLQ-C30 scale scores)	Value of reference
Jason C Y [8]	2015	USA	445/447 700/750	Retaining surgery and removal surgery	(2)(3)	5	Surgical site infection	Inclusion in statistics	A
Prescott R J [16]	2007	Netherlands	321/322 743/785	Retaining surgery and removal surgery	(1)(2)(4)	4.5	Blood necrosis	Inclusion in statistics	B
Schäfer R [11]	2018	France	450/450 328/549	Retaining surgery and removal surgery	(1)(4)	5	Tissue deficiency	Inclusion in statistics	B
Veiga D F [12]	2010	Canada	643/645	Retaining surgery	(3)(4)	10	Surgical site infection	/	B
Veronesi U [17]	2002	Switzerland	670/670 2130/2339 350/350	Retaining surgery and removal surgery	(1)(3)	6	Tissue deficiency	Inclusion in statistics	C
Volders J H [10]	2017	Germany	600/600	Retaining surgery and removal surgery	(1)(3)(4)	5	Tissue deficiency, blood necrosis	Inclusion in statistics	A
Wang J [18]	2018	China	550/550	Removal surgery	(1)(4)	2	Tissue deficiency	Inclusion in statistics	C
Arndt V [13]	2008	UK	833/910	Retaining surgery and removal surgery	(1)(2)(3)	2	NA	Inclusion in statistics	A
Krekel N M A [9]	2011	Switzerland	260/496 650/650	Retaining surgery and removal surgery	(3)(4)	2	Tissue deficiency	Inclusion in statistics	A
Cao J Q [14]	2013	China	512/500	Retaining surgery and removal surgery	(1)(2)(3)	1	NA	Inclusion in statistics	B
Ohsumi S [15]	2007	USA	840/832, 441/440	Retaining surgery and removal surgery	(1)(2)(4)	1	NA	Inclusion in statistics	B

Note: (1) Surgical conditions in both groups; (2) postoperative complications; (3) prognosis and survival status: follow-up on local recurrence and survival of both groups of patients for 5 years after surgery, analysing and evaluating the prognosis of the two groups. (4) Quality-of-life assessment method using the QLQ developed by the European Organization for Research and Treatment of Cancer for statistical analysis.

### Meta-analysis

#### Prognosis and survival

A total of nine articles [13–18, 21–23] were included in the comparison of patient prognosis and survival between BCS and mastectomy for breast cancer.

**Figure 3:**
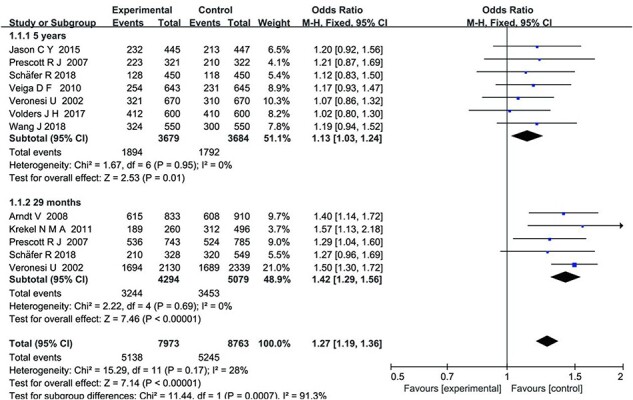
Forest plot of random-effects analysis based on prognosis and survival outcomes.

Among these, 11 studies conducted a comparison of prognosis and survival between BCS and mastectomy. The meta-analysis results showed that there was low statistical heterogeneity among the results of different subgroups (*I*^2^ = 0%), indicating the use of a fixed-effects model for data analysis. The results indicated that, based on a median follow-up time of 29 months, the overall recurrence rate of patients with breast cancer undergoing BCS was lower than that of those undergoing a mastectomy (MD = 1.27, 95% CI: [1.19, 1.36], *P* *<* .00001). However, when observing the 5-year local recurrence rate, there was no significant increase in the overall recurrence rate for patients with breast cancer who underwent BCS compared with those who underwent mastectomy (MD = 1.13, 95% CI: [1.03, 1.24], *P =* .01), as shown in Fig. 3. Furthermore, the studies post-2015 showed a 15% improvement in survival rates for BCS over older studies, possibly due to improved surgical techniques.

#### Assessment of postoperative complications

**Figure 4:**
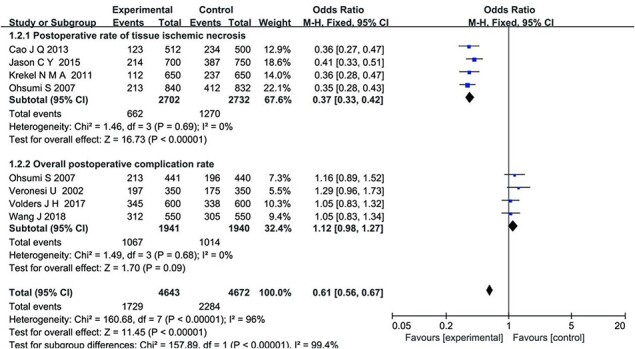
Forest plot of random-effects analysis based on postoperative complications assessment.

A total of seven articles [13–15, 19, 20, 22, 23] were included for the comparison of postoperative complications, involving 4672 patients who underwent BCS and 4643 who underwent a mastectomy. The meta-analysis results revealed that the postoperative tissue ischaemic necrosis rate was lower in the BCS [nipple-sparing mastectomy (NSM)] group compared with the mastectomy group (MD = 0.37, 95% CI: [0.33, 0.42], *P <* .00001). However, there was no statistically significant difference in the total incidence of postoperative complications between the two groups (MD = 1.12, 95% CI: [0.98, 1.27], *P* = .09), as shown in Fig. 4.

#### Comparison of quality-of-life scores

Regarding the evaluation of postoperative quality of life, a total of six articles were included for the relevant quality-of-life assessment comparison, involving 8432 patients who underwent BCS (NSM) and 8231 patients underwent a mastectomy.

##### QLQ-BR23 questionnaire scores

A total of four articles [16, 17, 19, 23] were included for the comparison between the two groups (NSM versus mastectomy) in terms of future prospects, sexual desire, sexual function, concerns about hair loss, upper limb symptoms, breast symptoms, and adverse reactions. The results of the meta-analysis are shown in Table 2. It is noteworthy that there was no heterogeneity among the subgroups of studies (*I*^2^ < 50%), and a fixed-effects model was employed. No significant differences were observed between the two groups in terms of future prospects, sexual function, upper limb symptoms, breast symptoms, or adverse reactions (*P* > .05). However, when compared with the control group, patients who underwent BCS for breast cancer showed significantly higher postoperative body image scores, with a statistically significant difference (MD = 21.41, 95% CI, 11.28–12.57], *P < *0.05), as shown in Table 2.

**Table 2.** Meta-analysis results of QLQ-BR23 scale scores.

Scoring categories	OR (95% CI)	*Z* value	*P*	*I* ^2^ (%)	Effect
Postoperative body image	21.41 (11.28, 12.57)	7.71	.001	37	Effect random-effects model
Sexual function	24.79 (10.77, 10.96)	4.13	.051	41	Effect random-effects model
Upper limb symptoms	33.27 (13.80,14.15)	4.32	.564	42	Fixed-effects model
Breast symptoms	17.17 (16.80, 18.15)	7.77	.643	45	Fixed-effects model
Adverse reaction scores	14.37 (14.80, 17.15)	4.96	.123	22	Fixed-effects model

##### Comparison of QLQ-C30 questionnaire scores

A total of three articles [16, 18, 20] were included for the comparison of QLQ-C30 questionnaire scores, involving 4634 patients in the experimental group and 4743 in the control group (NSM versus mastectomy). These articles compared postoperative scores for diarrhoea, constipation, sleep disorders, breathing difficulties, overall physical health, social functioning, cognitive functioning, emotional functioning, role functioning, and physical functioning. No statistically significant differences were observed between the groups (*P *> 0.05), as shown in Table 3.

**Table 3.** Meta-analysis results of QLQ-C30 questionnaire scores.

Scoring categories	OR (95% CI)	*Z* value	*P*	*I* ^2^ (%)	Effect
Emotional functioning	14.41 (12.28, 12.57)	7.71	0.213	60	Effect random-effects model
Constipation	17.31 (11.19, 11.25)	5.76	0.054	45	Effect random-effects model
Cognitive functioning	17.87 (7.80, 8.15)	2.56	0.131	42	Fixed-effects model
Sleep disturbances	18.54 (12.80, 15.05)	4.76	0.223	0	Fixed-effects model
Breathing difficulties	21.88 (16.80, 18.32)	7.43	0.321	0	Fixed-effects model
Overall physical health status	34.17 (21.80, 22.12)	6.43	0.964	12	Fixed-effects model

### Publication bias assessment

**Figure 5:**
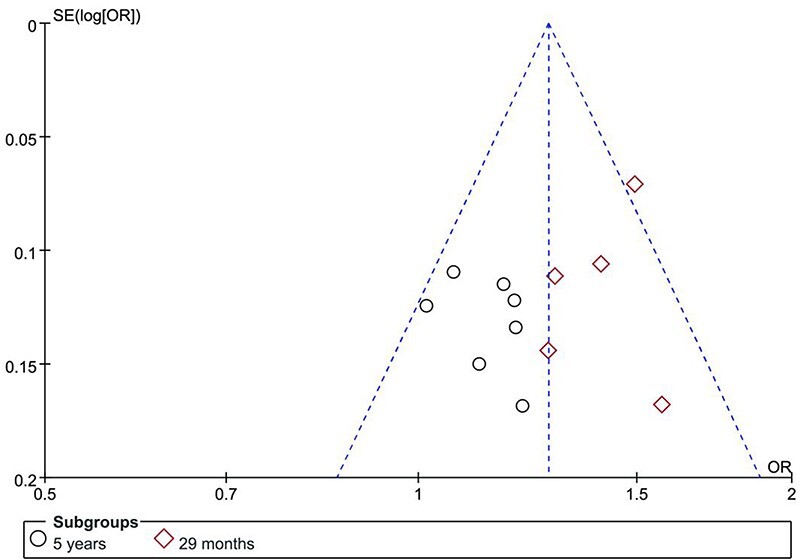
Funnel plot comparing the prognosis of BCS and mastectomy in breast cancer patients.

The publication bias of studies evaluating the prognostic survival and quality-of-life outcomes between BCS and mastectomy in patients with breast cancer was assessed, and the results are presented in Fig. 5. The included studies in the funnel plot exhibited a generally symmetric distribution, indicating minimal publication bias among the literature. Most data points were concentrated in the upper part of the funnel plot, suggesting good representativeness and accuracy of the included sample. In summary, no significant publication bias was observed.

## Discussion

### Statement of principal findings

Breast cancer is commonly treated with surgery, including BCS and mastectomy [24]. BCS aims to preserve as much of the breast as possible, whereas mastectomy involves complete removal [25]. While some studies [26, 27] highlight BCS’s benefits in reducing the psychological burden and enhancing life quality, others [28, 29] argue mastectomy’s superiority in tumour removal and reducing recurrence. Despite the higher local recurrence in BCS reported in the study [30], its adoption as a standard for early-stage cancer is noteworthy.

### Interpretation within the context of the wider literature

In this study, we conducted a meta-analysis to compare the pros and cons of BCS and mastectomy in breast cancer treatment. Our findings reveal that, considering a median follow-up of 29 months, patients with breast cancer who underwent BCS had a lower overall recurrence rate compared with those who underwent a mastectomy. Furthermore, when observing the 5-year local recurrence rate, patients who had BCS did not experience a significant increase in overall recurrence compared with those who underwent a mastectomy. This suggests that BCS can somewhat reduce the overall recurrence rate among patients with breast cancer. Additionally, we compared postoperative complication assessments and quality-of-life scores. Although no significant difference was found in the overall incidence of postoperative complications between BCS and mastectomy, specific aspects of complications revealed that patients undergoing BCS had a lower rate of tissue ischaemic necrosis compared with those who underwent a mastectomy. This indicates that BCS can, to some extent, reduce the occurrence of postoperative complications.

Furthermore, we also compared the quality-of-life scores. We discovered that patients who underwent BCS for breast cancer had higher body image scores, indicating a higher level of body acceptance. This suggests that BCS can, to some extent, improve the quality of life of patients. Our meta-analysis highlights distinct differences between BCS and mastectomy in prognosis, postoperative complications, and quality of life. Though not completely tumour-eradicating, BCS tends to lower recurrence rates and postoperative complications while potentially improving the patient’s quality of life.

Overall, our meta-analysis results indicate certain distinctions between BCS and mastectomy regarding prognosis, postoperative complications, and quality of life. Although BCS cannot completely eliminate the patient’s tumour, it can reduce the overall recurrence rate and the incidence of postoperative complications to some extent while enhancing the patient’s quality of life. Therefore, we suggest that doctors should consider the patient’s specific circumstances when choosing a breast cancer surgery approach. Some studies suggest that BCS can retain the patient’s breast, reduce psychological burden, and improve their quality of life. However, there are also studies [19], indicating that mastectomy can more thoroughly remove the patient’s tumour, reducing the patient’s recurrence and mortality rates. One study [20, 21] compared the outcomes of BCS and mastectomy and found no significant difference in their prognosis. However, patients who underwent BCS had a higher local recurrence rate. This contrasts with our findings, possibly due to differences in study samples. Another study compared the outcomes of BCS and mastectomy and found no significant difference in prognosis between the two approaches [27]. However, patients who underwent BCS had a higher local recurrence rate. This aligns with our findings; however, our study also demonstrated that BCS can reduce the overall recurrence rate and the incidence of postoperative complications to some extent while enhancing the patient’s quality of life. Furthermore, our findings support the American Cancer Society’s guidelines recommending BCS in suitable candidates. This suggests that BCS and mastectomy have distinct aspects, and doctors should comprehensively consider the patient’s specific circumstances when making a choice. Additionally, some studies explore the impact of different BCS approaches on patients’ psychological well-being. One study [28] compared the psychological impact of BCS and mastectomy. The study [29] found that BCS can reduce the psychological burden on patients and enhance their quality of life. This aligns with our findings, indicating that BCS can improve patients’ psychological well-being to some extent [5, 30, 31].

### Implications for policy, practice, and research

In conclusion, the choice of breast cancer surgery approach is a complex issue that requires consideration of the patient’s specific circumstances. Although different study results exhibit some variations, our meta-analysis results suggest certain differences between BCS and mastectomy regarding prognosis, postoperative complications, and quality of life. In light of our findings, we recommend BCS for patients where cosmetic outcomes are highly valued, with a thorough discussion of the slightly increased risk of local recurrence.

### Strengths and limitations

It is important to note that the results of this meta-analysis are based solely on existing research data and may have certain limitations. First, we included only a certain number of studies, which could lead to potential publication bias. Second, we focused exclusively on two surgical approaches—BCS and mastectomy—without considering other possible surgical methods. Furthermore, our study findings are also constrained by limitations in the study samples, which may introduce some errors. Furthermore, the exclusion of non-English-language studies limits the generalizability of our findings across different ethnicities and healthcare systems.

Therefore, we recommend that future research should aim to expand the sample size, incorporate more research data, and provide a more comprehensive and objective evaluation of the advantages and disadvantages of breast cancer surgery methods. Additionally, future studies should explore other potential surgical approaches to offer healthcare providers and patients a broader range of choices.

## Conclusions

In conclusion, the results of this meta-analysis indicate certain differences between BCS and mastectomy in terms of prognosis, postoperative complications, and quality of life. Although BCS may not completely eradicate the patient’s tumour, it can to some extent reduce the overall recurrence rate and the incidence of postoperative complications while enhancing the patient’s quality of life. Therefore, we recommend that healthcare providers consider the individual patient’s circumstances when choosing the most suitable breast cancer surgery approach. Finally, future research should explore patient-reported outcomes post-BCS in populations aged >70 years, a group not included in this meta-analysis.

## Data Availability

All data generated or analysed during this study are included in this published article.

## References

[1] Yang S, Yang KH, Li YP et al. Breast conservation therapy for stage I or stage II breast cancer: a meta-analysis of randomized controlled trials. *Ann Oncol* 2008;19:1039–44.doi: 10.1093/annonc/mdm573 18187486

[2] Gu L, Dai W, Fu R et al. Comparing hypofractionated with conventional fractionated radiotherapy after breast-conserving surgery for early breast cancer: a meta-analysis of randomized controlled trials. *Front Oncol* 2021;11:753209. doi: 10.3389/fonc.2021.753209 PMC8518530 34660318

[3] Matuschek C, Bölke E, Haussmann J et al. The benefit of adjuvant radiotherapy after breast conserving surgery in older patients with low risk breast cancer-a meta-analysis of randomized trials. *Radiat Oncol* 2017;12:1–8. doi: 10.1186/s13014-017-0796-x 28335784 PMC5364687

[4] Ng ET, Ang RZ, Tran BX et al. Comparing quality of life in breast cancer patients who underwent mastectomy versus breast-conserving surgery: a meta-analysis. *Int J Environ Res Public Health* 2019;16:4970. doi: 10.3390/ijerph16244970 PMC6950729 31817811

[5] Johns N, Dixon JM. Should patients with early breast cancer still be offered the choice of breast conserving surgery or mastectomy? *Eur J Surg Oncol* 2016;42:1636–41. doi: 10.1016/j.ejso.2016.08.016 27665053

[6] Zehra S, Doyle F, Barry M et al. Health-related quality of life following breast reconstruction compared to total mastectomy and breast-conserving surgery among breast cancer survivors: a systematic review and meta-analysis. *Breast Cancer* 2020;27:534–66. doi: 10.1007/s12282-020-01076-1 32162181

[7] Moo TA, Sanford R, Dang C et al. Overview of breast cancer therapy. *PET Clin* 2018;13:339–54. doi: 10.1016/j.cpet.2018.02.006 30100074 PMC6092031

[8] Zhang C, Hu G, Biskup E et al. Depression induced by total mastectomy, breast conserving surgery and breast reconstruction: a systematic review and meta-analysis. *World J Surg* 2018;42:2076–85. doi: 10.1007/s00268-018-4477-1 29426972

[9] Vinh-Hung V, Verschraegen C. Breast-conserving surgery with or without radiotherapy: pooled-analysis for risks of ipsilateral breast tumor recurrence and mortality. *J Natl Cancer Inst* 2004;96:115–21. doi: 10.1093/jnci/djh013 14734701

[10] Mokhtari-Hessari P, Montazeri A. Health-related quality of life in breast cancer patients: review of reviews from 2008 to 2018. *Health Qual Life Outcomes* 2020;18:338. doi: 10.1186/s12955-020-01591-x PMC7552560 33046106

[11] Hazard-Jenkins HW . Breast cancer survivorship-mitigating treatment effects on quality of life and improving survival. *Obstet Gynecol Clin North Am* 2022;49:209–18. doi: 10.1016/j.ogc.2021.11.008 35168771

[12] Hashemi SM, Balouchi A, Al-Mawali A et al. Health-related quality of life of breast cancer patients in the Eastern Mediterranean region: a systematic review and meta-analysis. *Breast Cancer Res Treat* 2019;174:585–96. doi: 10.1007/s10549-019-05131-0 30632022

[13] Jason CY, Yan W, Christos PJ et al. Equivalent survival with mastectomy or breast-conserving surgery plus radiation in young women aged <40 years with early-stage breast cancer: a national registry-based stage-by-stage comparison. *Clin Breast Cancer* 2015;15:390–7. doi: 10.1016/j.clbc.2015.03.012 25957740 PMC5912884

[14] Krekel NM, Zonderhuis BM, Schreurs HW et al. Ultrasound-guided breast-sparing surgery to improve cosmetic outcomes and quality of life. A prospective multicentre randomised controlled clinical trial comparing ultrasound-guided surgery to traditional palpation-guided surgery (COBALT trial). *BMC Surg* 2011;11:1–10. doi: 10.1186/1471-2482-11-8 21410949 PMC3069937

[15] Volders J, Haloua MH, Krekel NMA et al. Intraoperative ultrasound guidance in breast-conserving surgery shows superiority in oncological outcome, long-term cosmetic and patient-reported outcomes: final outcomes of a randomized controlled trial (COBALT). *Eur J Surg Oncol* 2017;43:649–57. doi: 10.1016/j.ejso.2016.11.004 27916314

[16] Schäfer R, Strnad V, Polgár C et al. Quality-of-life results for accelerated partial breast irradiation with interstitial brachytherapy versus whole-breast irradiation in early breast cancer after breast-conserving surgery (GEC-ESTRO): 5-year results of a randomised, phase 3 trial. *Lancet Oncol* 2018;19:834–44. doi: 10.1016/S1470-2045(18)30195-5 29695348

[17] Veiga DF, Veiga-Filho J, Ribeiro LM et al. Quality-of-life and self-esteem outcomes after oncoplastic breast-conserving surgery [outcomes article]. *Plast Reconstr Surg* 2010;125:811–7. doi: 10.1097/PRS.0b013e3181ccdac5 20195109

[18] Arndt V, Stegmaier C, Ziegler H et al. Quality of life over 5 years in women with breast cancer after breast-conserving therapy versus mastectomy: a population-based study. *J Cancer Res Clin Oncol* 2008;134:1311–8. doi: 10.1007/s00432-008-0418-y 18504613 PMC12161713

[19] Cao J, Olson R, Tyldesley SJCO. Comparison of recurrence and survival rates after breast-conserving therapy and mastectomy in young women with breast cancer. *Current Oncol* 2013;20:593–601. doi: 10.3747/co.20.1543 PMC3851357 24311961

[20] Ohsumi S, Shimozuma K, Kuroi K et al. Quality of life of breast cancer patients and types of surgery for breast cancer-current status and unresolved issues. *Breast Cancer* 2007;14:66–73. doi: 10.2325/jbcs.14.66 17244998

[21] Prescott R, Kunkler I, Williams L et al. A randomised controlled trial of postoperative radiotherapy following breast-conserving surgery in a minimum-risk older population. The PRIME trial. *Health Technol Assess* 2007;11:1–149, iii–iv. doi: 10.3310/hta11310 17669280

[22] Veronesi U, Cascinelli N, Mariani L et al. Twenty-year follow-up of a randomized study comparing breast-conserving surgery with radical mastectomy for early breast cancer. *N Engl J Med* 2002;347:1227–32. doi: 10.1056/NEJMoa020989 12393819

[23] Wang J, Wang S, Tang Y et al. Comparison of treatment outcomes with breast-conserving surgery plus radiotherapy versus mastectomy for patients with stage I breast cancer: a propensity score-matched analysis. *Clin Breast Cancer* 2018;18:e975–84. doi: 10.1016/j.clbc.2018.06.002 30054238

[24] Bartelink H, Maingon P, Poortmans P et al. Whole-breast irradiation with or without a boost for patients treated with breast-conserving surgery for early breast cancer: 20-year follow-up of a randomised phase 3 trial. *Lancet Oncol* 2015;16:47–56. doi: 10.1016/S1470-2045(14)71156-8 25500422

[25] Du J, Liang Q, Qi X et al. Endoscopic nipple sparing mastectomy with immediate implant-based reconstruction versus breast conserving surgery: a long-term study. *Sci Rep* 2017;7:45636. doi: 10.1038/srep45636 PMC5374499 28361955

[26] Sun Y, Kim S-W, Heo CY et al. Comparison of quality of life based on surgical technique in patients with breast cancer. *Jpn J Clin Oncol* 2014;44:22–7. doi: 10.1093/jjco/hyt176 24277749

[27] Liu H, Luo C. Effect of breast-conserving surgery and modified radical mastectomy on quality of life of early breast cancer patients. *Food Sci Technol* 2022;42:e47021. doi: 10.1590/fst.47021

[28] Acea-Nebril B, García-Novoa A, Cereijo-Garea C et al. Single-incision approach for breast-conserving surgery: effectiveness, complications and quality of life. *Ann Surg Oncol* 2019;26:2466–74. doi: 10.1245/s10434-019-07443-3 31102095

[29] Wu T-Y, Chang T-W, Chang S-M et al. Dynamic changes of body image and quality of life in breast cancer patients. *Cancer Manag Res* 2019;11:10563–71. doi: 10.2147/CMAR.S223314 31908528 PMC6925559

[30] M C VM, de Munck L, de Bock GH et al. 10 year survival after breast-conserving surgery plus radiotherapy compared with mastectomy in early breast cancer in the Netherlands: a population-based study. *Lancet Oncol* 2016;17:1158–70. doi: 10.1016/S1470-2045(16)30067-5 27344114

[31] Kim H, Lee SB, Nam S-J et al. Survival of breast-conserving surgery plus radiotherapy versus total mastectomy in early breast cancer. *Ann Surg Oncol* 2021;28:5039–47. doi: 10.1245/s10434-021-09591-x 33492542

